# Circular RNA: new star, new hope in cancer

**DOI:** 10.1186/s12885-018-4689-7

**Published:** 2018-08-20

**Authors:** Zikang Zhang, Qing Xie, Dongmei He, Yuan Ling, Yuchao Li, Jiangbin Li, Hua Zhang

**Affiliations:** 1 Guangdong Provincial Key Laboratory of Medical Molecular Diagnostics, Institute of Laboratory Medicine, Guangdong Medical University, Dongguan, 523808 China; 2 Department of Gynaecology and Obstetrics, Huizhou Hospital of Traditional Chinese Medicine, Huizhou, 516000 China

**Keywords:** Circular RNA, MicroRNA sponge, Cancer, Regulation network, Exosome, Clinical implication

## Abstract

**Background:**

Circular RNAs are a new class of endogenous non-coding RNA that can function as crucial regulators of diverse cellular processes. The diverse types of circular RNAs with varying cytogenetics in cancer have also been reported.

**Main body of the abstract:**

Circular RNAs can act as a microRNA sponge or through other mechanisms to regulate gene expression as either tumor inhibitors or accelerators, suggesting that circular RNAs can serve as newly developed biomarkers with clinic implications. Here, we summerized recent advances on circular RNAs in cancer and described a circular RNA network associated with tumorigenesis. The clinical implications of circular RNAs in cancer were also discussed in this paper.

**Short conclusion:**

Growing evidence has revealed the crucial regulatory roles of circular RNAs in cancer and the elucidation of functional mechanisms involving circular RNAs would be helpful to construct a circRNA-miRNA-mRNA regulatory network. Moreover, circular RNAs can be easily detected due to their relative stability, widespread expression, and abundance in exosomes, blood and saliva; thus, circular RNAs have potential as new and ideal clinical biomarkers in cancer.

## Background

More than 75% of non-coding RNAs have been found in transcription of the human genome [1]. Circular RNAs (circRNAs), 100 bp to 4 kb in size, were regarded as non-functional by-products of aberrant RNA splicing [2, 3]. Recently, with the improvements in novel next-generation deep sequencing and bioinformatics technology, an increasing number of circRNAs with regulatory functions were found in many tapes of cancers [4–6]. Unlike linear transcripts, the structures of circRNAs are covalently closed loops without tails in the 5′-3′ port, which stabilizes the structures enough to resist digestion by RNase [7–10]. CircRNAs are generally classified as three types: exonic circRNAs, exonic-intronic circRNAs and intronic circRNAs. Most exonic circRNAs exist in cytoplasm, whereas the other two are mainly found in cell nucleus [10]. Some circRNAs exist in human body fluid, making them easy to be detected [9, 10]. Most circRNAs are extremely abundant, relatively stable and widely expressed in eukaryotic cells, suggesting that circRNAs have potential regulatory roles [11].

Some circRNAs discovered in human tissues have been related to diverse cellular processes, including senescence, growth and apoptosis, etc. [12, 13]. Moreover, deregulated circRNA expression profiles correlated with some cancers have been identified, suggesting that circRNAs can function as tumor inhibitors or accelerators [14]. Emerging evidence that circRNAs are important regulators in cancer implies they might serve as new clinical biomarkers in cancer [15, 16]. This review concentrates on recent advances in circRNA research in cancer and summarizes the current significance of circRNAs in the clinical implications of cancer.

## The regulation mechanisms of circRNAs

**Fig. 1:**
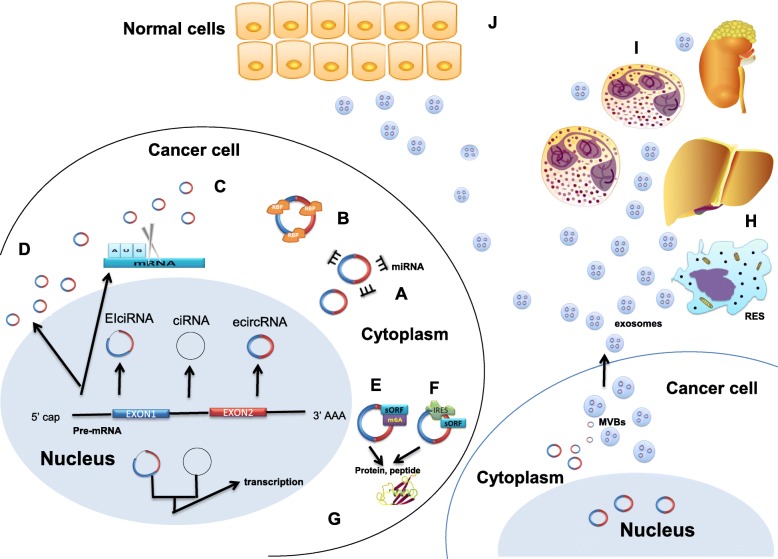
The regulation of circRNAs in cancer. **a**. CircRNAs function as microRNA (miRNA) sponges. **b**. CircRNAs bind RNA binding proteins (RBPs) as transcription regulators. **c**. CircRNAs remove start codons from mature mRNAs to reduce protein translation. **d**. CircRNA production competes with canonical pre-mRNA splicing in gene regulation. **e**. The translation of circRNAs is driven by N^6^-methyladenosine (m^6^A). **f**. CircRNAs with internal ribosome entry sites (IRESs) can be translated. **g**. EIciRNAs and ciRNAs promote transcription. **h**. Exo-circRNAs are cleared by the RES or excreted via the liver or kidneys. **i.** Exo-circRNAs regulate cancer immunity. **j**. Exo-circRNAs promote cancer metastasis

The regulation mechanisms of circRNAs have been revealed by increasing studies. The most notable of these mechanisms is that circRNAs can work as microRNA (miRNA) sponges. CircRNAs can block the binding of miRNAs with the 3’ UTR of a specific gene by directly binding to miRNAs, thus indirectly regulating the gene expression [17, 18]. For example, ciRS-7 can function as a sponge of miR-7 and consequently repress its function in cancer [19]. The second mechanism is that circRNAs play as regulators in gene expression by competing with mRNA production in pre-mRNA splicing [20]. Another mechanism of circRNAs involves binding to RNA binding proteins (RBPs) as transcription regulators [15, 16]. Moreover, circRNAs can serve as mRNA traps, another form of alternative splicing, and remove start codons from mature mRNAs to reduce protein translation in cancer [21] (Fig. 1).

### Translation of circRNAs

Translation of ncRNAs is poorly noted due to the classic ORFs longer than 100 codon are lacking. With more research on small open reading frames (sORFs), the proteins or peptides with biological functions that are translated by ncRNAs have received more attention [22]. CircRNAs, as a novel form of ncRNAs described in recent studies, have been found to be abundantly expressed in the cytoplasm, suggesting that they have the potential to regulate disease processes via translation of proteins or peptides [23]. Four mechanisms of circRNA protein or peptide translation have been identified. Wang et al. found that artificial synthetic circRNAs with internal ribosome entry sites (IRESs) can be translated [24]. Another mechanism has been found that circRNAs were effectively translated according to roll circle amplification (RCA) in human liver cells [25]. In addition, Yun et al. found that translation of circRNAs was driven by N^6^-methyladenosine (m^6^A) in human cells [26]. Recent research has found a novel cap-independent translation mechanism in circRNAs [22]. There have also been exciting breakthroughs in the study of circRNA protein translation as it relates to the regulation of cancer progression. FBXW7-185aa, which is translated from circ-FBXW7, regulates the stability of c-myc and inhibits the development of malignant glioma. This suggests that the functional protein resulting from circRNA translation may be a biomarker or therapeutic target for cancer. This demonstrates a new regulation mechanism of circRNAs in cancer [27] (Fig. 1).

### Exosome delivery of circRNAs in cancer

Extracellular vesicles released by cells can be divided into three categories according to origin and size, including microvesicles, apoptotic bodies, and exosomes [28]. Many biological molecules exist in EVs, such as DNA, RNA, bioactive lipids, and proteins [28, 29]. Exosomes are approximately 30 to 100 nm in diameter and can be derived from many cells; in addition, exosomes can be transported from the originating cell to the recipient cell [30]. Exosomes with coding transcripts and non-coding RNAs are easily discovered in accessible body fluids, particularly blood, and are released more frequently by tumor cells, implying that exosomes can act as cancer communication agents to help cancer cells escape from immune surveillance and contribute to tumor formation [30, 31].

Recently, a number of studies have found that more exosomes are released from cancer cells than from normal cells. It was reported that circRNAs in gastric cancer (GC) can be transferred from GC cells to normal cells via exosomes, indicating that exo-circRNAs are important in the peritoneal metastasis of GC [32]. Moreover, deregulated circRNAs have been found in the exosomes of different cancers, and cancer-associated chromosomal translocations generate fusion-circRNAs-exosomes that can promote cellular transformation and tumor progression [33, 34]. Interestingly, other studies have also found that exosomes can participate in the clearance of intracellular circRNAs, and exosomes themselves can be further cleared by the reticuloendothelial system (RES) or excreted via the liver or kidneys [35, 36] (Fig. 1).

### Expression profiles and identification of circRNAs in cancer

As microarray chip and next-generation sequencing technologies have been developed, many circRNAs were examined or identified in cancer samples. The expression profiles of circRNAs during the early stages of pancreatic ductal adenocarcinoma (PDAC) have been demonstrated, which revealed that deregulated circRNAs may participate in the progression of PDAC and potentially serve as a novel therapeutic biomarker [37, 38]. In another study, microarray analysis also showed that circRNA_100855 and circRNA_104912 are the most significantly deregulated circRNAs in laryngeal cancer tissues, whereas circRNA_001059 and circRNA_000167 are significantly deregulated in radioresistant esophageal cancer [39, 40]. In colorectal cancer, 379 dysregulated circRNAs were identified using circRNA microarray analysis [41].

In gliomas, RNA-Seq data showed the existence of over 476 deregulated circRNAs [42]. A recent study identified circRNAs associated with breast cancer subtypes using Circ-Seq [43]. Additionally, circRNA expression profiles in KRAS mutant colon cancer were identified from RNA-Seq data [44].

Interestingly, by combining microarray circRNA expression profiles with bioinformatics target prediction and sequence analysis, many deregulated circRNAs with miRNA response elements (MREs) have been identified in basal cell carcinoma (BBC) and cutaneous squamous cell carcinoma (CSCC) [45, 46]. More recently, it was reported that 69 differentially expressed circRNAs might interact with certain miRNAs to influence mRNA expression in gastric cancer (GC) [47].

### The circRNA regulation network in cancer

**Fig. 2:**
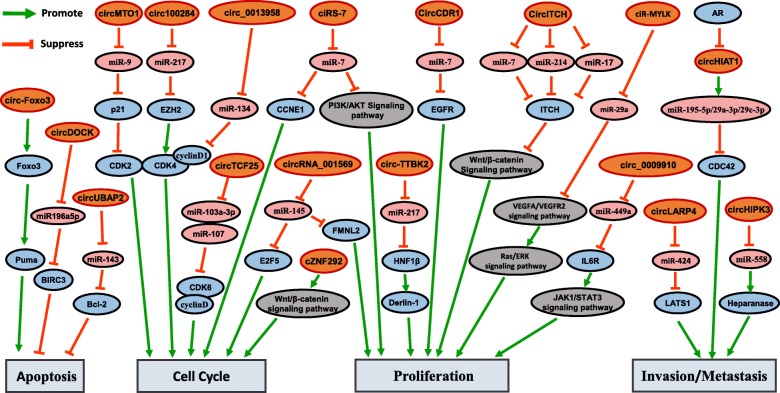
Regulation network of circRNAs in cancer. CircRNA can act as sponge of miRNA or combine with protein to indirectly regulate gene expression in cancer. Circ-Foxo3, circDOCK and circUBAP2 are involved in regulating apoptosis in cancer. CircMTO1, circ100284, hsa_circ_0013958 and circTCF25 can regulate cell cycle through indirectly regulating cell cycle proteins in cancer. CiRS-7, circITCH, ciR-MYLK, circ-TTBK2 and circ_0009910 are involved in cell proliferation partly through PI3K/AKT pathway, Wnt/β-catenin pathway VEGFA/VEGFR2 pathway and JAK1/STAT3 pathway, respectively. CircHIAT1, circLARP4 and circHIPK3 are involved in invasion or metastasis through indirectly regulating CDC42, LATS1, and Heparanase in cancer, respectively. Different colores indicate different types of molecules: orange represents circRNA, pink represents miRNA, blue represents coding gene, gray represents signaling pathway

Although the overall mechanisms of circRNAs in cancers have not been entirely elucidated, crucial regulatory roles of circRNAs in cancer have been revealed. Recent studies on circRNAs mainly focused on the roles as miRNA sponges, interactions with binding proteins and translation into proteins or peptides [22, 48]. An increasing amount of evidence has shown the involvement of circRNAs in regulatory signaling pathways that influence the progression and development of cancer,making circRNAs a potential therapeutic target [49]. Here, a clearer circRNA regulation network in cancer and its relevance to tumorigenesis is summarized (Fig. 2).

CircRNAs regulate apoptosis in cancer. It has been shown that circ-Foxo3 can promote MDM2-induced degradation of p53 by binding to MDM2 and p53; however, circ-Foxo3 contributes more to repression of MDM2-induced Foxo3 ubiquitination by binding to Foxo3 and thus increasing the expression of the downstream gene PUMA to induce apoptosis in breast carcinoma [50, 51]. In another study, increased circUBAP2 was found to upregulate its target Bcl-2 and inhibit apoptosis in osteosarcoma by sponging miR-143 [52].

CircRNAs regulate the cell cycle in cancer. It has been demonstrated that miR-7 can inhibit cancer progression by suppressing CCNE1 and PIK3CD in hepatocellular carcinoma (HCC) [53, 54]. A recent study proved that ciRS-7 can function as an oncogene to halt the cell cycle by upregulating CCNE1 and promoting cell proliferation via PI3K/AKT pathway by directly targeting miR-7 in HCC [55]. Moreover, deregulated miR-217 can target EZH2, which can increase the level of cyclin D1 to accelerate cell cycle progression and lead to malignant transformation. In addition, upregulated circ100284 can bind miR-217 and promote cell cycle progression in arsenic-induced skin cancer [56].

CircRNAs regulate cancer proliferation. The overall expression of circ-CDR1 can also increase EGFR expression and lead to cell proliferation by sponging miR-7 in HCC [57]. Additionally, it was revealed that circ-ITCH can inhibit miR-7 to partly enhance the effect of ITCH, which suppresses cell proliferation by inhibiting the Wnt/β-Catenin pathway in lung cancer and ESCC [58, 59]. In bladder cancer, another study demonstrated that circTCF25 can suppress miR-107 and miR-103a-3p to accelerate proliferation and migration, which led to increased CDK6 and further activation of cyclin D to promote cell cycle progression into the S phase [60].

CircRNAs regulate invasion and metastasis in cancer. Upregulated androgen receptor (AR) expression can accelerate the development of clear cell renal cell carcinoma (CCRCC) by inhibiting miR-145 [61]. Recently, a new mechanism of AR regulation was revealed. AR can enhance migration and invasion through circHIAT1-microRNA-protein signaling, and circHIAT1 can increase signaling by serving as a miRNA suppressor more so than a miRNA sponge in CCRCC [62]. Previous studies demonstrated that E2F5 can promote cell growth and is frequently observed in diverse human cancers [63]. Furthermore, overexpression of circ_001569 accelerates proliferation and invasion through targeting miR-145, which suppresses E2F5 and FMNL2 in colorectal cancer (CRC) [64]. In addition, it was reported that circHIPK3 competes with miR-558 to inhibit heparinase and cause rapid invasion metastasis in bladder cancer [65].

Although the rough roles of several circRNAs in some cancers have been confirmed, the functions and regulation pathways of most circRNAs in cancer remain to be revealed.

### Clinically relevant implications of circRNAs in cancer

Differential expression profiling analysis and functional studies of circRNAs in tumors are important for the further understanding of circRNAs and cancer. Meanwhile, similar to microRNAs and lncRNAs, circRNAs also show potential as new independent diagnostic and prognostic biomarkers, which provides new approaches to improve clinical diagnosis and treatment. Here, we summarize currently known cancer-associated circRNAs related to clinical implications in Table 1 and mainly discuss the potential of some circRNAs as clinical biomarkers.

**Table 1:** Cancer associated circRNAs

Cancer type	CircRNA	Samples	Cases	Expression	Association	Reference
Bladder cancer	CircPTK2	Tissue/blood	40 pairs	Up	Poor differentiation, higher lymph node metastasis and T stage	[83]
	Circ-ITCH	Tissue	72 pairs	Down	Higher TNM stage and histological grade	[96]
Esophageal cancer	Has_circ_0067934	Tissue	51 pairs	Up	Poor differentiation and higher TNM stage	[84]
	CiRS-7	Tissue	86 pairs	Up	Higher clinical stage and pathological grade	[97]
Colorectal cancer	Hsa_circ_0007534	Tissue	33 pairs	Up	Higher clinical stage and lymph node metastasis	[68]
	Hsa_circ_001988	Tissue	31 pairs	Down	Associated with differentiation and perineural invasion	[67]
	Hsa_circ_0000069	Tissue	30 pairs	Up	Associated with patient age and TNM stage	[98]
	CiRS-7	Tissue	40 pairs	Up	Higher T-stage and metastasis/poor prognosis	[70]
	Circular BANP	Tissue	35 pairs	Up	Unknown	[41]
	CircRNA0003906	Tissue	122 pairs	Down	Poor differentiation, higher lymphatic metastasis/diagnosis value	[99]
	Hsa_circ_0001649	Tissue/blood	Total 146	Down (tissue)/up (blood)	Associated with differentiation	[100]
	Circ_0014717	Tissue	46 pairs	Down	Associated with TNM stage and distal metastasis/poor prognosis	[69]
	Hsa_circ_0000567	Tissue	102 pairs	Down	Lower clinical stage and lymph node metastasis/diagnosis value	[101]
	CircHIPK3	Tissue	Total 218	Up	Associated with TNM stage and metastasis/poor prognosis	[102]
Hepatocellular carcinoma	CiRS-7	Tissue	108 pairs	Up (39.8%)/down (60.2%)	Associated with MVI	[103]
	Hsa_circ_0005075	Tissue	66 pairs	Up	Larger size tumors/diagnostic potential	[75]
	Hsa_circ_0001649	Tissue	89 pairs	Down	Larger tumor size and tumor embolus formation/poor prognosis	[104]
	Hsa_circ_0003570	Tissue	107 pairs	Down	Associated with tumor diameter, differentiation and vascular formation	[73]
	CircMTO1	Tissue	261 pairs	Down	Poor prognosis	[74]
	CircZKSCAN1	Tissue	102 pairs	Down	Potential diagnostic value	[76]
	Hsa_circ_0000673	Tissue	51 pairs	Up	Poor overall survival	[105]
	CircC3P1	Tissue	47 pairs	Down	Higher TNM stage, tumor size and vascular invasion	[106]
Gastric cancer	Hsa_circ_002059	Tissue/plasma	Total 147	Down	Associated with distal metastasis, TNM stage, gender and age	[11]
	Hsa_circ_0000190	Tissue/plasma	104 pairs	Down	Associated with tumor diameter, metastasis and TNM stage (tissue)/CEA (plasma)	[81]
	Circ-104916	Tissue	70 pairs	Down	Higher tumor stage and lymphatic metastasis	[80]
	CircRNA_100269	Tissue	112 pairs	Down	Associated with histological subtypes and nodes invasion	[107]
	Hsa_circ_0000745	Tissue/plasma	20 pairs	Down	Associated with tumor differentiation (tissue)/node metastasis (plasma)	[108]
	Hsa circ 0074362	Tissue	127 pairs	Down	Associated with CA19–9 and lymphatic metastasis	[109]
	CircPVT1	Tissue	187 pairs	Up	Associated with overall survival	[110]
	Hsa_circ_0006633	Tissue/plasma	Total 338	Down(tissue)/up(plasma)	Associated with distal metastasis and CEA (tissue)	[78]
	Hsa_circ_0001895	Tissue	Total 257	Down	Associated with differentiation, Borrmann type and CEA	[111]
	Hsa_circ_0014717	Tissue/gastric juice	Total 122	Down	Associated with tumor stage, metastasis, CEA and CA19–9 (tissue)	[79]
	Hsa_circ_0003159	Tissue	108 pairs	Down	Higher gender, distal metastasis and node metastasis	[112]
	Hsa_circ_0000181	Tissue/plasma	Total 115	Down	Associated with tumor diameter, metastasis and CA19–9 (tissue)/differentiation and CEA	[82]
	Hsa_circ_0000520	Tissue	56 pairs	Down	Higher TNM stage	[113]
	CircMYO9B	Tissue	21 pairs	Up	Lower survival rate	[114]
Breast cancer	CircGFRA1	Tissue	Total 222	Up	Higher tumor size, TNM stage, lymphatic metastasis and histological grade	[115]
	Cir-ITCH	Tissue	Total 78	Up	Associated with age	[58]
	CircRNA_100876	Tissue	101 pairs	Up	Associated with lymphatic metastasis and advanced stage	[116]
Osteosarcoma	CircPVT1	Tissue/serum/lung metastasis	Total 80	Up	Poor prognosis/diagnosis value	[95]
Lung cancer	CircFADS2	Tissue	43 pairs	Up	Poor differentiation, advanced TNM stage and lymphatic metastasis	[117]
	CircRNA_102231	Tissue	57 pairs	Up	Associated with TNM stage and lymph node metastasis	[118]

### CircRNAs in colorectal cancer

Colorectal cancer has become the fourth most deadly cancer in the world, and its occurrence is related to changes in individual genetics [66]. CircRNAs might potentially be a new biomarker to facilitate CRC diagnosis and prognosis. A positive correlation between several deregulated circRNAs in CRC and clinical indicators has been identified. For example, qRT-PCR analysis of 31 CRC patients showed that circ_001988 expression is downregulated and significantly associated with peripheral invasion and less differentiation [67]. Additionally, higher expression of hsa_circ_0007534 in CRC tumor tissue is associated with neoplasm staging and lymphatic metastasis [68]. CircRNAs may be used to predict prognosis in CRC, as Wang et al. found that patients with downregulated hsa_circ_0014717 have poorer OS and poor prognosis [69]. Moreover, overexpressed ciRS-7 can promote aggressiveness of CRC and is positively related with a high T-stage and lymphatic and distant metastasis, implying that ciRS-7 might be releated to a worse prognosis [70].

### CircRNAs in hepatocellular carcinoma

HCC is responsible for nearly 90% of primary malignancies of the liver, and patients with advanced stage disease always have poor prognoses [71, 72]. CircRNAs might function as a prognostic predictor and therapeutic target in HCC. One study demonstrated that downregulated circ_0003570 is closely related to tumor diameter, differentiation status and vascular formation in HCC [73]. Another study revealed that downregulated circMTO1 is associated with dismal prognosis in HCC and that upregulated circMTO1 can act as a sponge of miR-9 to increase the level of p21 and inhibit the malignant development of HCC [74]. Moreover, upregulated circ_0005075 is correlated with larger tumor size and increased cell adhesion, whereas downregulated circZKSCAN1 is involved in several cancer-related signaling pathways to suppress the growth of HCC. Both of the AUROCs for these circRNAs indicated a potential diagnostic value [75, 76].

### CircRNAs in gastric cancer

Although many efforts have been made to improve the diagnosis and therapy of GC, five-year OS rates in gastric cancer patients are still less than 30% [77]. New biomarkers for diagnosis and therapy are still necessary, and up to now, mostly low expression of circRNAs in GC has been observed. The clinical samples have been derived from not only tumor tissue and plasma but also gastric juice, suggesting that circRNAs may be useful potential biomarkers [78, 79]. The downregulated circ-104916 has been found to be associated with higher invasion, neoplasm staging and lymph node metastasis in GC [80]. Additionally, both circ_0000190 and circ_002059 are more lowly expressed in GC tissues, which is relevant to some clinical parameters [11, 81]. Furthermore, the expression of circ_0000181 is significantly decreased in GC, and circ_0000181 is associated with many clinical indicators in GC patients, implying that it might serve as a good biomarker [82].

### CircRNAs in other cancers

In breast cancer, downregulated circ-Foxo3 can enhance cell survival and decrease cell apoptosis [52]. In bladder cancer, circPTK2 is highly expressed among fourty pairs of tissues and blood specimens, and its expression level is significantly associated with lower differentiation, N2-N3 lymphatic metastasis, and higher T stage [83]. In ESCC, circ_0067934 is significantly upregulated and associated with poor differentiation, whereas cir-ITCH is downregulated and functions as a tumor inhibitor by regulating tumor cell viability [59, 84]. In lung cancer and colorectal cancer, downregulated circ-ITCH plays an important tumor suppressor role [58, 85]. Upregulated circSMARCA5 can accelerate cell cycle and suppress apoptosis in prostate cancer [86]. In glioma, both circ-TTBK2 and cZNF292 are highly expressed and play crucial oncogenic roles in promoting glioma malignancy progression [87, 88].

### CircRNAs in chemoradiation resistance

The emergence of chemoradiation resistance can lead to poor prognosis or recurrence [89–91]. At present, studies have found changed circRNA expression profiles in radioresistant ESCC cells, ADM-resistant breast cancer cells and 5-FU-based chemoradiation-resistant CRC cells. Through biological analysis, some circRNAs have been found to influence the chemoradiation resistance of cancer cells by regulating specific genes or pathways [40, 92–94]. Another study has revealed that downregulated circPVT1, which is overexpressed in osteosarcoma tissues and chemoradiation-resistant cells, can weaken expression of the classical chemoradiation resistance gene ABCB1 to reduce the resistance to cisplatin and doxorubicin in osteosarcoma cells [95]. Although there is still very little research regarding circRNAs and chemoradiation resistance in cancer, it has a great potential that circRNAs can be used as novel biomarkers to predict the efficiency of chemoradiation and prognosis or recurrence in drug-resistant cancers.

## Conclusions

Many studies indicated that circRNAs, similar to miRNAs and lncRNAs, may have significant regulatory effects on pathophysiologic processes, including tumorigenesis. The connections of circRNAs with cancer has become a hot research field. CircRNAs can be easily detected due to their relative stability, widespread expression, and abundant presence in exosomes, blood and saliva, indicating that circRNAs might be novel and ideal diagnostic and prognostic biomarkers in cancer. In this paper, we drew conclusions about recent advances on circRNAs in cancer and presented a circRNA-mediated network involved in cell cycle control, apoptosis, proliferation, invasion and metastasis in cancer.

At present, some circRNA expression profiles in several cancers have been identified; however, there are still many questions that need to be addressed. Further investigation is needed regarding the various gene regulatory mechanisms of circRNAs other than miRNA sponges. The important relationship between the exo-circRNAs and tumor metastasis and the development of novel and valid ways to predict target genes of circRNAs using bioinformatics, among other issues, need to be addressed. This will provide new insights into circRNAs to construct circRNA-miRNA-mRNA regulation networks, reveal cancer pathogenesis mechanisms and seek novel potential diagnosis biomarker or therapeutic targets for future cancer management.

## Abbreviations

Akt/PKB: v-akt murine thymoma viral oncogene homolog 1/protein kinase B
AR: Androgen receptor
BBC: Basal cell carcinoma
BC: Breast cancer
CCNE1: Cyclin E1
CCRCC: Clear cell renal cell carcinoma
CDK2: Cyclin-dependent kinase2
CDK4: Cyclin-dependent kinase4
CDK6: Cyclin-dependent kinase6
circRNAs: Circular RNAs
CRC: Colorectal cancer
ESCC: Esophageal cancer
EVs: Extracellular vesicles
GC: Gastric cancer
HCC: Hepatocellular carcinoma
miRNA: microRNA
mTOR: Mammalian target of rapamycin
MVI: Microvascular invasion
ncRNA: Non-coding RNA
OS: Overall survival
PDAC: Pancreatic ductal adenocarcinoma
PIK3CD: Phosphoinositide 3-kinase catalytic subunit delta
RBP: RNA binding proteins
RES: Reticuloendothelial system
RT-PCR: Reverse transcription PCR
